# Evaluation of Regional Body Circumferences and Serum Albumin for Identifying Nutritional Risk in Gastric Adenocarcinoma

**DOI:** 10.3390/medicina62102023

**Published:** 2026-10-19

**Authors:** Oğuzhan Kesen, Neşe Alyıldız, Esra Aydın, Sıtkı Utku Akay, Ali Çelik

**Affiliations:** 1 Department of Medical Oncology, Faculty of Medicine, Recep Tayyip Erdoğan University, Rize 53020, Türkiye; oguzhan.kesen@erdogan.edu.tr (O.K.);; 2 Department of Medical Oncology, Recep Tayyip Erdoğan University Training and Research Hospital, Rize 53020, Türkiye; 3 Department of Radiation Oncology, Muğla Sıtkı Koçman University Training and Research Hospital, Muğla 48000, Türkiye; 4 Department of Emergency Medicine, Faculty of Medicine, Recep Tayyip Erdoğan University, Rize 53020, Türkiye

**Keywords:** gastric adenocarcinoma, nutritional risk, NRS-2002, arm circumference, serum albumin, anthropometry

## Abstract

*Background and Objectives:* We evaluated the association of regional body circumferences, body mass index (BMI), and serum albumin with concurrent nutritional risk in gastric adenocarcinoma. *Materials and Methods:* This single-center retrospective cross-sectional study included patients assessed between January 2024 and January 2026. Nutritional risk was defined as a Nutritional Risk Screening 2002 (NRS-2002) score ≥ 3. Receiver operating characteristic analysis assessed discrimination of this screening outcome. Logistic models incorporating arm circumference alone or with albumin were evaluated in the same complete-case sample using 1000 bootstrap resamples for internal validation. *Results:* Among 102 patients, 100 had NRS-2002 scores and 20 (20.0%) had nutritional risk. In 95 complete cases, including 19 patients at risk, areas under the curve (AUCs) were 0.869 for arm circumference, 0.864 for BMI, and 0.858 for albumin. In the combined model, each 1-cm decrease in arm circumference and each 0.1-g/dL decrease in albumin were associated with higher odds of nutritional risk: mutually adjusted odds ratios were 1.67 (95% confidence interval [CI], 1.22–2.29) and 1.27 (95% CI, 1.08–1.51), respectively. Adding albumin improved model fit (*p* = 0.001). Exploratory, internally validated optimism-corrected AUCs were 0.926 for the combined model and 0.873 for arm circumference alone. *Conclusions:* Arm circumference and albumin were associated with NRS-2002-defined nutritional risk. The small number of outcome events, incomplete covariate information, and absence of external validation limit interpretation. These findings do not establish a diagnosis of malnutrition, improvement in screening, or clinical utility.

## 1. Introduction

Gastric cancer continues to impose a substantial burden on global health. According to the GLOBOCAN 2022 estimates, approximately 968,000 new cases and 660,000 deaths were attributed to gastric cancer worldwide, placing it fifth among cancers for both incidence and mortality [1]. Nutritional deterioration is an important concern in the care of these patients. In cancer, inadequate intake may coexist with metabolic disturbances related to the disease, while surgery and systemic treatments can introduce further challenges to maintaining nutritional status. These changes have implications for patients’ quality of life and their ability to tolerate treatment, making nutritional care relevant throughout the course of oncological management [2].

Recognition of nutritional problems requires an approach that extends beyond a single measurement obtained at presentation. The European Society for Clinical Nutrition and Metabolism (ESPEN) recommends evaluating dietary intake, changes in body weight, and body mass index (BMI) from the time of cancer diagnosis, with reassessment according to the patient’s clinical condition. When screening identifies abnormalities, a more detailed evaluation should address the factors limiting intake, muscle mass, physical performance, and systemic inflammation [2]. Within this broader assessment process, simple measurements that can be obtained during routine clinical encounters may help characterize nutritional vulnerability and provide additional context for interpreting screening results.

The Nutritional Risk Screening 2002 (NRS-2002) instrument considers both the patient’s nutritional condition and the nutritional consequences of disease. Its scoring system incorporates indicators of nutritional impairment, including reduced intake, weight loss, and low BMI, together with disease severity and an adjustment for older age. A total score of at least 3 identifies nutritional risk. The instrument was developed around the relationship between nutritional impairment, illness severity, and the potential benefit of nutritional support [3]. Consequently, an association between an individual measurement and NRS-2002 reflects that measurement’s relationship with a composite screening outcome, rather than with an isolated feature of nutritional depletion.

This distinction is relevant when defining study outcomes. The Global Leadership Initiative on Malnutrition (GLIM) proposes a sequential process in which screening is followed by assessment to establish a diagnosis. Malnutrition requires the coexistence of at least one phenotypic criterion, such as involuntary weight loss, low BMI, or reduced muscle mass, and at least one etiologic criterion, involving reduced intake or assimilation, or disease burden and inflammation [4]. The 2025 GLIM update retained this central diagnostic structure [5]. Accordingly, studies using NRS-2002 as their reference should describe their outcome as nutritional risk and interpret their findings within the scope of screening.

BMI remains useful because it is familiar, easily calculated, and widely available. Nevertheless, patients with similar BMI values may differ considerably in their body composition. In a cohort of 1473 patients with lung or gastrointestinal cancers, Martin et al. demonstrated substantial variation in muscle characteristics across BMI categories and found that computed tomography (CT)-defined muscle depletion and low muscle attenuation were associated with survival [6]. These findings indicate that body weight relative to height does not fully describe tissue reserves. They also provide a rationale for considering measurements that contribute information beyond overall body size when evaluating nutritional vulnerability in cancer.

Arm circumference is a practical anthropometric measurement that requires minimal equipment and can be obtained at the bedside. Earlier ESPEN screening guidance recognized mid-arm circumference as a useful alternative when reliable height and weight measurements cannot be obtained [7]. Its value is primarily accessibility: the measured circumference reflects the combined dimensions of muscle, subcutaneous fat, bone, and other tissues and can also be affected by fluid accumulation. It therefore provides a nonspecific indication of body size and tissue reserves rather than a direct measurement of muscle mass. CT-based assessment can characterize muscle more directly, but requires suitable imaging and additional analysis. The present study did not compare tape measurements with CT or validate them as measures of sarcopenia. Abdominal and thigh circumferences were examined as additional regional measures in relation to the same nutritional screening outcome.

Serum albumin provides another readily available measurement, although its interpretation differs from that of anthropometry. The American Society for Parenteral and Enteral Nutrition (ASPEN) emphasizes that albumin and other visceral proteins are influenced by inflammation and should not be used as proxies for total body protein or muscle mass. Their association with adverse outcomes does not make them specific indicators of malnutrition [8]. Albumin may therefore contribute information about the broader clinical context. Whether this information improves recognition of nutritional risk when added to arm circumference is a question that requires direct evaluation.

Recent studies in gastric cancer have explored the combination of measurements from different domains. Huang et al. evaluated a model integrating CT-derived psoas muscle features with clinical variables, including BMI and albumin, using NRS-2002 as the reference outcome [9]. Xian et al. investigated an index combining mid-arm circumference with an albumin–prealbumin–globulin ratio in relation to nutritional assessment and survival [10]. A related question for routine care is how simple tape measurements compare within the same patient sample and whether a single laboratory measurement adds information about concurrent nutritional risk.

We therefore evaluated the associations of arm, abdominal, and thigh circumferences, BMI, and serum albumin with baseline nutritional risk, defined as NRS-2002 ≥ 3, within a cohort of patients with gastric adenocarcinoma. We compared discrimination of this outcome and explored the internally validated performance of models incorporating arm circumference alone or with albumin.

## 2. Materials and Methods

### 2.1. Study Design and Population

This single-center retrospective cross-sectional study examined adults who attended the Department of Medical Oncology, Recep Tayyip Erdoğan University Training and Research Hospital, Rize, Türkiye, between January 2024 and January 2026. The analytic cohort comprised 102 patients with gastric adenocarcinoma or compatible histological descriptions in the study records. Eligible descriptions included intestinal, diffuse, signet-ring cell, mucinous, tubular, poorly differentiated, poorly cohesive, and mixed forms. Their distribution is reported in Supplementary Table S3. These categories summarize the recorded descriptions rather than a centrally reassessed pathological classification. Gastrointestinal stromal tumors, neuroendocrine neoplasms, other mesenchymal tumors, and records with insufficient information to establish histological or primary-site eligibility were excluded. Each patient contributed one baseline assessment, defined as the first available nutritional assessment in the study records. Although some patients underwent a three-month reassessment, only the first assessment was used in this analysis. Patients with missing individual measurements remained in the overall cohort and contributed to analyses for which the required variables were available.

### 2.2. Clinical and Nutritional Assessment

Age, sex, height, body weight, weight loss during the preceding 3 months, arm circumference, abdominal circumference, thigh circumference, serum albumin, and nutritional screening scores were obtained from clinical records. Circumferences were measured using a tape and expressed in centimeters. Body mass index (BMI) was calculated as weight in kilograms divided by height in meters squared. Three-month weight loss was expressed in kilograms and as a percentage of previous body weight, calculated as 100 × weight lost/(current weight + weight lost). Serum albumin was expressed in g/dL.

Circumference measurements were obtained by the same dietitian for all patients and at each assessment visit, with the patient standing. Arm and thigh circumferences were measured on the left side. Exact anatomical landmarks, tape tension, and whether measurements were repeated within the same visit could not be retrospectively verified.

Data availability reflected the completeness of the retrospective study records. Patient-specific reasons for missing NRS-2002, albumin, or three-month weight-loss information could not be established. Recorded NRS-2002 total scores were used as documented; an unavailable separate weight-loss field did not imply that this component was absent when screening was performed. CRP, tumor stage, metastatic status, treatment timing, edema, and ascites were not available in a sufficiently complete and standardized form for analysis. Some records contained free-text surgical and treatment information, but these entries did not support reliable classification at the baseline nutritional assessment.

Nutritional risk was assessed using the NRS-2002 total score, which combines nutritional impairment and disease severity with an age adjustment for patients aged 70 years or older [7]. The primary outcome was NRS-2002 ≥ 3; scores < 3 defined the comparison group. NRS-2002 served as a reference for nutritional risk, not a diagnostic reference standard for malnutrition. Arm circumference was the principal anthropometric measure of interest; abdominal circumference, thigh circumference, BMI, and albumin were evaluated as additional measures. All associations concerned concurrent nutritional risk at baseline.

### 2.3. Statistical Analysis

Continuous variables are presented as medians with interquartile ranges (IQRs), and categorical variables as counts and percentages. Groups were compared using the two-sided Mann–Whitney U test for continuous variables and Fisher’s exact test for sex. The prevalence of NRS-2002 ≥ 3 was estimated with an exact binomial 95% confidence interval (CI). Spearman rank correlations were used to assess associations between NRS-2002 scores and the five measures of interest.

Patients with missing NRS-2002 were excluded from outcome-based analyses, while measurement-specific analyses used available observations. Paired AUC comparisons and both logistic models used a common complete-case sample. We additionally summarized age, sex, anthropometry, albumin, and observed nutritional risk according to completeness of the main analysis variables and availability of weight loss, albumin, and NRS-2002. These comparisons were descriptive; small incomplete-data groups limited inference about the missing-data mechanism.

Discrimination of NRS-2002 ≥ 3 was assessed using receiver operating characteristic (ROC) curves and the area under the curve (AUC), with 95% CIs calculated by the DeLong method. Measure-specific analyses used all available observations. Direct comparisons of arm circumference with BMI and albumin used paired DeLong tests in a common sample with complete data for all five measures. These tests evaluated evidence of a difference, not equivalence or noninferiority. Exploratory thresholds were selected by maximizing the Youden index, with sensitivity, specificity, positive predictive value, and negative predictive value estimated in the same sample. These threshold estimates were not externally validated or separately corrected for the optimism introduced by selecting the threshold.

The Benjamini–Hochberg procedure was applied separately to the 10 continuous-variable group comparisons, five NRS-2002 correlations, and five univariable logistic analyses. Holm correction was used for the two paired AUC comparisons. All tests were two-sided, with *p* < 0.05 considered statistically significant; adjusted *p* values were used for test families subject to multiplicity correction.

Univariable logistic regression estimated associations with NRS-2002 ≥ 3. Odds ratios (ORs) and Wald 95% CIs were expressed per 1-cm decrease in arm or thigh circumference, 5-cm decrease in abdominal circumference, 1-kg/m^2^ decrease in BMI, and 0.1-g/dL decrease in albumin. To limit model complexity with only 19 outcome events, two models were evaluated in the same complete-case sample: arm circumference alone and arm circumference with albumin. Continuous terms were modeled linearly on the log-odds scale, with no interaction terms or stepwise variable selection. The combined model estimated two predictor coefficients plus an intercept, corresponding to 9.5 outcome events per predictor coefficient. This ratio was descriptive and was not taken as proof of sufficient sample size or model stability. Age and sex were not included in these deliberately limited models; the reported ORs are adjusted for the other measurement only. Tumor stage, treatment timing, and inflammatory status could not be included because sufficiently complete, standardized information was unavailable for analysis. The contribution of albumin was assessed using a likelihood-ratio test.

Model performance was assessed using AUC, Brier score, calibration slope, and calibration-in-the-large. Internal validation used 1000 patient-level bootstrap resamples. Both models were refitted in each resample and evaluated in the resampled and original data. Optimism-corrected estimates subtracted the mean training-minus-original-sample difference from apparent performance. Paired replicates were retained when both model fits satisfied the convergence and numerical validity criteria, and a corrected metric was reported only when at least 95% of resamples yielded valid estimates. This procedure estimated optimism within the development cohort and did not evaluate performance in independent patients. All corrected performance estimates were interpreted as exploratory.

A sensitivity analysis repeated the analyses in patients with an explicit adenocarcinoma diagnosis, applying a narrower histological eligibility definition. Statistical analyses were performed using R version 4.6.1, with the pROC package version 1.19.1 for ROC analyses [11].

### 2.4. Use of Generative Artificial Intelligence

OpenAI Codex (version 26.1007.30912; accessed September and October 2026) assisted with R code development and language editing.

### 2.5. Ethics

The study was approved by the Recep Tayyip Erdoğan University Health Sciences Ethics Committee on 1 September 2026 (decision number 2026/172). Informed consent was waived because of the retrospective study design.

## 3. Results

### 3.1. Study Population

A total of 122 patients with records indicating possible gastric malignancy were assessed for eligibility. Twenty patients were excluded because of gastrointestinal stromal tumors (n = 8), neuroendocrine neoplasms (n = 3), other mesenchymal tumors (n = 1), a confirmed non-gastric primary tumor (n = 3), or insufficient histological information to establish eligibility for the gastric adenocarcinoma cohort (n = 5). The analytic cohort included 102 patients, of whom 75 (73.5%) were men. Median age was 58.5 years (IQR, 50.0–66.0). Of these, 100 had NRS-2002 scores: 80 had scores < 3 and 20 had scores ≥ 3, corresponding to a nutritional risk prevalence of 20.0% (95% CI, 12.7–29.2%). Two patients without NRS-2002 were excluded from outcome-based analyses. Albumin was missing in five additional patients, leaving 95 complete cases with 19 patients at risk for the paired AUC comparisons and logistic models. Three-month weight loss was available for 65 patients; this variable was not required for entry into the main model. Baseline characteristics are shown in Table 1, recorded histological descriptions in Supplementary Table S3, and characteristics according to data availability in Supplementary Table S4.

Compared with the 95 complete cases, the seven patients outside the common analysis had a median age of 65.0 versus 58.0 years and median arm circumference of 27.0 versus 26.0 cm; five were men. One of the five with an available NRS-2002 score had nutritional risk. Among patients with observed NRS-2002, nutritional risk was present in 19/63 (30.2%) with recorded three-month weight loss and 1/37 (2.7%) without it. Median BMI was 22.5 and 25.6 kg/m^2^, respectively, in the weight-loss-available and weight-loss-missing groups (Supplementary Table S4).

### 3.2. Characteristics According to Nutritional Risk

Patients with NRS-2002 ≥ 3 were older than those with NRS-2002 < 3 (median, 67.0 vs. 56.0 years; adjusted *p* = 0.019). They had lower body weight, BMI, arm circumference, abdominal circumference, thigh circumference, and serum albumin (all adjusted *p* < 0.001). Median arm circumference was 22.0 versus 27.0 cm, BMI was 18.6 versus 24.1 kg/m^2^, and albumin was 3.20 versus 3.87 g/dL. Percentage weight loss over 3 months was greater in the NRS-2002 ≥ 3 group (14.0% vs. 8.2%; adjusted *p* = 0.001). Sex distribution, height, and absolute weight loss did not differ significantly between groups (Table 1).

### 3.3. Correlations and Discrimination

NRS-2002 scores correlated inversely with arm circumference (Spearman ρ = −0.455), abdominal circumference (ρ = −0.405), thigh circumference (ρ = −0.506), BMI (ρ = −0.539), and albumin (ρ = −0.520). All correlations remained significant after adjustment for multiple comparisons (all adjusted *p* <0.001; Table 2).

In the common sample of 95 patients, AUCs were 0.869 (95% CI, 0.780–0.958) for arm circumference, 0.864 (0.783–0.945) for BMI, 0.858 (0.781–0.934) for albumin, 0.795 (0.679–0.911) for thigh circumference, and 0.776 (0.665–0.887) for abdominal circumference. Paired comparisons did not detect a difference between arm circumference and BMI (AUC difference, 0.005; 95% CI, −0.063 to 0.072) or albumin (0.011; −0.107 to 0.129); both Holm-adjusted *p* values were 1.000 (Table 2). These results do not establish equivalence between the measures.

### 3.4. Logistic Regression and Model Performance

Lower values of all five measures were associated with higher odds of NRS-2002 ≥ 3 in univariable analyses (all adjusted *p* < 0.001; Table 3). In the two-variable model, a 1-cm decrease in arm circumference was associated with an OR of 1.67 (95% CI, 1.22–2.29; *p* = 0.002), and a 0.1-g/dL decrease in albumin with an OR of 1.27 (1.08–1.51; *p* = 0.005), each adjusted for the other measure. Adding albumin improved model fit compared with arm circumference alone (likelihood-ratio χ^2^ = 10.11, df = 1; *p* = 0.001).

The arm-plus-albumin model had an apparent AUC of 0.930, compared with 0.869 for the arm-only model. Exploratory, internally validated optimism-corrected AUCs were 0.926 and 0.873, respectively. Corresponding corrected Brier scores were 0.095 and 0.103, and calibration slopes were 0.950 and 1.022. These estimates used 954 valid paired bootstrap resamples; 46 of 1000 resamples did not meet the paired model-fit criteria. Corrected calibration intercepts had 937 valid estimates for the combined model and 947 for the arm-only model, below the required threshold, and are not reported (Table 4). These results quantify internal performance and do not establish clinical validity in an independent population.

### 3.5. Sensitivity Analysis and Exploratory Thresholds

The histology sensitivity analysis included 92 patients with an explicitly documented adenocarcinoma diagnosis, of whom 90 had NRS-2002 data and 18 had scores ≥ 3 (20.0%; 95% CI, 12.3–29.8%). The 10 patients omitted from this narrower definition had compatible descriptions without an explicit adenocarcinoma term: six had signet-ring cell descriptions, two intestinal-type descriptions, one a diffuse-type description, and one a poorly cohesive carcinoma description (Supplementary Table S3). Among 86 complete cases, the AUC was 0.860 for arm circumference, 0.864 for BMI, and 0.882 for albumin. Arm circumference and albumin retained their mutually adjusted associations, with ORs of 1.56 (95% CI, 1.15–2.12) and 1.35 (1.10–1.65), respectively. The combined model had an exploratory, internally validated optimism-corrected AUC of 0.929, based on 994 valid bootstrap resamples (Supplementary Table S1). This sensitivity analysis used a subset of the same cohort and was not an external validation.

Exploratory Youden thresholds are shown in Supplementary Table S2. In the main cohort, arm circumference < 22.5 cm identified 11 of 20 patients with NRS-2002 ≥ 3 (sensitivity, 55.0%) and yielded 77 true-negative classifications among 80 patients with NRS-2002 < 3 (specificity, 96.3%). Positive and negative predictive values were 78.6% and 89.5%, respectively. Nine of 20 patients at risk were missed. These are apparent estimates from the same sample used to select the threshold.

## 4. Discussion

In this cohort of patients with gastric adenocarcinoma, one in five patients with an available NRS-2002 score was at nutritional risk. Lower arm circumference and albumin were associated with higher odds of NRS-2002 ≥ 3 after adjustment for the other measure. Arm circumference had an AUC of 0.869 in the common analysis sample; paired comparisons did not detect differences from BMI or albumin and did not establish equivalence. Adding albumin improved model fit and yielded an exploratory, internally validated optimism-corrected AUC of 0.926.

The observed nutritional risk prevalence of 20.0% was lower than the 43.5% reported by Yang and Yang in 294 surgical patients with gastrointestinal cancers [12]. Their cohort combined gastric and colorectal cancers, assessed risk before surgery, and included patients receiving neoadjuvant chemotherapy. Such differences in eligibility, tumor burden, treatment exposure, and timing of assessment limit direct comparison with a medical oncology cohort. Measurements at diagnosis, after surgery, or during systemic treatment may capture different nutritional states. The direction or magnitude of these influences could not be established in our patients because stage and treatment timing were not adequately characterized. Differences between screening instruments and diagnostic assessments also matter: NRS-2002-defined risk, GLIM-defined malnutrition, and PG-SGA classifications represent related but distinct outcomes. Our estimate should therefore be interpreted as the proportion with NRS-2002 ≥ 3 in this selected cohort, rather than a general prevalence of malnutrition in gastric cancer [4,5].

The inverse associations between circumferences and NRS-2002 scores are consistent with lower anthropometric reserves among patients at nutritional risk. Prior work has shown why body composition may add information to weight-based assessment: Martin et al. identified clinically relevant muscle depletion across BMI categories [6], and Rimini et al. linked early CT-measured muscle loss during chemotherapy with survival in metastatic gastric cancer [13]. In a Turkish cohort of 177 patients with metastatic gastric cancer, Kanbur et al. also found associations between CT-defined low muscle mass, inflammatory indices, and the prognostic nutritional index [14]. These studies provide a rationale for assessing body composition, but a tape circumference includes contributions from both muscle and fat. Our findings cannot establish sarcopenia, for which consensus assessment includes muscle strength and confirmation of low muscle quantity or quality [15].

The study by Xian et al. offers a related comparison with our combined model. Among 1803 patients with gastric cancer, the authors evaluated mid-arm circumference together with the albumin–prealbumin–globulin ratio and reported associations with survival and severe malnutrition defined by the Patient-Generated Subjective Global Assessment (PG-SGA) [10]. Our model uses albumin alone with arm circumference and addresses NRS-2002-defined risk at the same assessment. Differences in laboratory measures, measurement protocols, and outcomes prevent direct comparison of performance estimates. Similarly, Huang et al. combined CT-derived features with BMI, lymphocyte count, and albumin using NRS-2002 as the reference [9]. The present results justify further study of a simpler combination; they do not show that it can replace CT-based assessment or established nutritional screening.

The contribution of albumin should be interpreted in the context of systemic illness. The ASPEN position paper emphasizes that serum albumin is influenced by inflammatory processes and should not be used as a surrogate for body protein or muscle mass [8]. In a gastric cancer surgical cohort, Matsui et al. found that the association between GLIM-defined malnutrition and survival varied according to preoperative inflammatory status [16]. Because CRP and other inflammatory measures were unavailable for adjustment in our analysis, the association of albumin with nutritional risk may partly reflect systemic inflammation or disease burden. Its nutritional and inflammatory contributions cannot be separated here. A 2026 multicenter study of patients receiving perioperative FLOT (fluorouracil, leucovorin, oxaliplatin, and docetaxel) found no significant association between the investigated inflammatory or nutritional indices and pathological response [17]. An association with concurrent NRS-2002-defined risk should therefore not be interpreted as evidence of treatment-response or survival prediction.

The reference outcome also limits interpretation. BMI and recent weight loss contribute to the nutritional impairment component of NRS-2002 [3,7]. Evaluating BMI or weight-related variables against this score therefore introduces incorporation bias: part of the information being evaluated is already contained in the reference outcome. This overlap can strengthen associations or discrimination without demonstrating independent identification of malnutrition. The score also includes an age adjustment from 70 years, so the observed age difference may partly reflect its construction. Arm circumference and albumin are not explicit score components, but association with the composite score still does not establish diagnostic validity against GLIM, PG-SGA, or a clinical nutritional assessment.

The exploratory arm circumference threshold is not supported as a stand-alone screening rule. At <22.5 cm, specificity was 96.3%, but sensitivity was 55.0%; nine of 20 patients with nutritional risk would have been missed. An arm circumference of 22.5 cm or greater cannot be used to rule out nutritional risk. The Youden index gives equal weight to sensitivity and specificity, which may not reflect the consequences of missing nutritional risk. Moreover, selecting and assessing a cutoff in the same dataset can overestimate its performance and produce an unstable threshold. Bootstrap correction of the logistic models did not validate this separate cutoff-selection procedure. The cutoff should therefore remain hypothesis-generating, with no recommendation for routine implementation. Regular screening and individualized assessment remain appropriate [2].

The common analysis sample and paired AUC comparisons supported consistent comparisons, and the narrower histological analysis yielded similar findings within the same cohort. Nevertheless, bootstrap internal validation estimates optimism within the development sample and cannot establish transportability to new patients or centers [18]. Only 19 outcome events supported the two predictor coefficients in the combined model. A descriptive ratio of 9.5 events per predictor does not establish model stability; sample size requirements also depend on the outcome frequency, anticipated model fit, and precision required [19]. Restricting model complexity and avoiding stepwise selection reduced the number of fitted parameters but did not remove small-sample uncertainty. The failure of 46 paired bootstrap fits and insufficient valid calibration-intercept estimates are additional reasons for caution. The models were not adjusted for age, sex, stage, or treatment, so mutually adjusted associations should not be described as independent of these factors.

Other limitations arise from the retrospective, single-center design and incomplete records. The extent of selection before inclusion in the analytic cohort could not be quantified. Seven patients lacked the variables required for the common analysis; they were older than complete cases in the descriptive comparison. Weight-loss availability also differed with nutritional risk: NRS-2002 ≥ 3 was present in 19/63 patients with recorded weight loss and 1/37 without it. These differences are compatible with selective documentation and preclude assuming that missingness was completely random, although they do not identify its mechanism. Weight-loss analyses may therefore be particularly susceptible to selection bias. Its absence did not exclude patients from the arm-plus-albumin model.

The cohort was predominantly male (75/102, 73.5%), and only six women with observed NRS-2002 had nutritional risk. The study therefore could not reliably establish sex-specific performance or thresholds. Differences in tissue distribution, body habitus, stage, and treatment may alter the relationship between circumference and nutritional risk. Edema may enlarge limb circumferences, and ascites may enlarge abdominal circumference without indicating greater tissue reserves. Prior gastrectomy and anticancer treatment may affect intake, body weight, tissue mass, and inflammatory activity; fluid balance and systemic inflammation can also affect serum albumin. Incomplete characterization of these factors leaves residual confounding and limits generalizability.

Although all measurements were performed by one dietitian, intraobserver repeatability was not assessed. Some technical details, including exact anatomical landmarks, could not be verified retrospectively. The analysis concerned one baseline assessment and did not evaluate subsequent nutritional change, treatment toxicity, or survival. A prospective external study should standardize measurement methods, characterize stage, treatment and fluid status, and assess performance across relevant patient groups. It should also include nutritional assessment using GLIM criteria or PG-SGA and determine whether adding these measures to existing care changes decisions or improves outcomes.

## 5. Conclusions

Lower arm circumference and serum albumin were associated with concurrent NRS-2002-defined nutritional risk in this gastric adenocarcinoma cohort. Their combination showed exploratory internal discrimination, with substantial limitations from the small number of outcome events, incomplete covariate information, and absence of external validation. The findings do not establish malnutrition diagnosis, equivalence between individual measures, or improvement in nutritional screening or clinical outcomes. Prospective external validation and evaluation of clinical utility are required before the model or exploratory thresholds can guide practice.

## Figures and Tables

**Table 1:** Baseline characteristics according to nutritional risk.

Characteristic	Overall N = 102	NRS-2002 < 3 n = 80	NRS-2002 ≥ 3 n = 20	*p*	BH-Adjusted *p*
Age, years	58.5 (50.0–66.0)	56.0 (49.0–65.3)	67.0 (54.5–72.5)	0.015	0.019
Male sex, n (%)	75 (73.5)	59 (73.8)	14 (70.0)	0.781	—
Female sex, n (%)	27 (26.5)	21 (26.3)	6 (30.0)	—	—
Height, cm	167.5 (159.0–172.0)	168.0 (160.0–172.0)	162.5 (157.0–172.0)	0.227	0.227
Weight, kg	64.2 (54.6–75.0)	67.1 (58.7–76.5)	50.0 (41.6–56.0)	<0.001	<0.001
BMI, kg/m^2^	23.0 (20.1–26.7)	24.1 (22.0–27.2)	18.6 (17.4–21.3)	<0.001	<0.001
Arm circumference, cm	26.0 (24.0–28.0)	27.0 (25.0–30.0)	22.0 (20.8–25.0)	<0.001	<0.001
Abdominal circumference, cm	87.5 (81.0–97.0)	90.5 (83.8–100.5)	78.5 (72.0–86.5)	<0.001	<0.001
Thigh circumference, cm	42.0 (38.0–45.0)	43.0 (39.8–46.0)	36.5 (33.0–40.3)	<0.001	<0.001
Albumin, g/dL	3.74 (3.34–4.05)	3.87 (3.50–4.17)	3.20 (2.89–3.45)	<0.001	<0.001
Weight loss over 3 months, kg	7.0 (5.0–10.0)	6.0 (3.8–10.0)	8.0 (6.0–10.0)	0.082	0.091
Weight loss over 3 months, %	10.2 (6.8–16.1)	8.2 (5.8–12.3)	14.0 (10.2–20.6)	<0.001	0.001

Values are median (IQR) or n (%). Group comparisons exclude the two patients with missing NRS-2002. Available observations in the overall, NRS-2002 < 3, and NRS-2002 ≥ 3 groups, respectively: albumin, 97, 76, and 19; weight loss, 65, 44, and 19. All other variables were complete. *p* values are from Mann–Whitney U tests for continuous variables and Fisher’s exact test for sex. BH adjustment covers the 10 continuous-variable comparisons. Percentages may not sum to 100% because of rounding. BH, Benjamini–Hochberg; BMI, body mass index; IQR, interquartile range; NRS-2002, Nutritional Risk Screening 2002.

**Table 2:** Correlations with NRS-2002 and discrimination of NRS-2002 ≥ 3.

(A) Correlations and AUC Estimates					
Measure	n/at Risk	ρ	BH-Adjusted *p*	AUC (95% CI) Available Cases	AUC (95% CI) Common Cases
Arm circumference	100/20	−0.455	<0.001	0.843 (0.744–0.942)	0.869 (0.780–0.958)
Abdominal circumference	100/20	−0.405	<0.001	0.778 (0.671–0.885)	0.776 (0.665–0.887)
Thigh circumference	100/20	−0.506	<0.001	0.805 (0.692–0.917)	0.795 (0.679–0.911)
BMI	100/20	−0.539	<0.001	0.868 (0.791–0.946)	0.864 (0.783–0.945)
Albumin	95/19	−0.520	<0.001	0.858 (0.781–0.934)	0.858 (0.781–0.934)
**(B) Paired AUC comparisons in the common sample**					
**Comparison**		**AUC Difference (95% CI)**		***p***	**Holm-Adjusted *p***
Arm vs. BMI		0.005 (−0.063–0.072)		0.896	1.000
Arm vs. albumin		0.011 (−0.107–0.129)		0.854	1.000

Spearman ρ describes the correlation with NRS-2002 score. BH adjustment covers the five correlation tests. Available-case analyses include 100 patients with 20 NRS-2002 ≥ 3 for anthropometric measures and 95 with 19 NRS-2002 ≥ 3 for albumin. All common-case AUC estimates and paired comparisons use the same 95 patients, including 19 with NRS-2002 ≥ 3. Lower values of each measure indicate greater nutritional risk. AUC confidence intervals and paired tests use the DeLong method. Differences are arm AUC minus comparator AUC. Holm adjustment covers the two paired comparisons. AUC, area under the receiver operating characteristic curve; BH, Benjamini–Hochberg; BMI, body mass index; CI, confidence interval. At risk denotes NRS-2002 ≥ 3.

**Table 3:** Logistic regression analyses of nutritional risk.

(A) Univariable Models Using All Available Cases					
Measure	Decrease	n/at Risk	OR (95% CI)	*p*	BH-Adjusted *p*
Arm circumference	1 cm	100/20	1.66 (1.30–2.12)	<0.001	<0.001
Abdominal circumference	5 cm	100/20	1.62 (1.24–2.12)	<0.001	<0.001
Thigh circumference	1 cm	100/20	1.30 (1.14–1.49)	<0.001	<0.001
BMI	1 kg/m^2^	100/20	1.61 (1.29–2.02)	<0.001	<0.001
Albumin	0.1 g/dL	95/19	1.36 (1.17–1.57)	<0.001	<0.001
**(B) Models fitted to the same 95 complete cases with 19 NRS-2002 ≥ 3**					
**Model**	**Term**		**OR (95% CI)**		***p***
Arm only	Arm, per 1-cm decrease		1.78 (1.35–2.35)		<0.001
Arm plus albumin	Arm, per 1-cm decrease		1.67 (1.22–2.29)		0.002
Arm plus albumin	Albumin, per 0.1-g/dL decrease		1.27 (1.08–1.51)		0.005

The outcome is NRS-2002 ≥ 3. ORs are expressed per indicated decrease, with Wald 95% CIs. BH adjustment covers the five univariable tests in Panel A. Panel B presents unadjusted *p* values. Panel B models use the same 95 patients, including 19 with NRS-2002 ≥ 3. In the two-variable model, arm circumference and albumin are adjusted for each other only. Adding albumin to arm circumference: likelihood-ratio χ^2^ = 10.11, df = 1, *p* = 0.001. BH, Benjamini–Hochberg; CI, confidence interval; OR, odds ratio. At risk denotes NRS-2002 ≥ 3.

**Table 4:** Apparent and exploratory internally validated model performance.

(A) Performance in the Main Cohort				
Metric	Arm only Apparent	Arm only Corrected	Arm plus Albumin Apparent	Arm plus Albumin Corrected
AUC	0.869	0.873	0.930	0.926
Brier score	0.100	0.103	0.088	0.095
Calibration slope	1.000	1.022	1.000	0.950
Calibration intercept	0.000	NR	0.000	NR
**(B) Number of valid bootstrap estimates**				
**Metric**		**Arm only**	**Arm plus Albumin**	
AUC		954/1000	954/1000	
Brier score		954/1000	954/1000	
Calibration slope		954/1000	954/1000	
Calibration intercept		947/1000	937/1000	

Both models use the same 95 patients, including 19 with NRS-2002 ≥ 3. Internal validation used 1000 patient-level bootstrap resamples, retaining paired replicates in which both model fits were valid. Corrected estimates subtract mean bootstrap optimism from apparent performance and are exploratory estimates within the development cohort, not externally validated performance. At least 950 valid estimates were required to report each corrected metric. Calibration intercepts did not meet this criterion (NR). Calibration intercept denotes calibration-in-the-large. Apparent intercepts and slopes are approximately 0 and 1 when evaluated in the development data. AUC, area under the receiver operating characteristic curve; NR, not reported.

## Data Availability

The data are not publicly available due to ethical considerations and national and institutional restrictions on data sharing. Access may be considered in exceptional, duly justified circumstances, where permitted by applicable policies and subject to the necessary ethical and institutional approvals.

## Supplementary Materials

The following supporting information can be downloaded at: https://www.mdpi.com/article/10.3390/medicina62102023/s1, Table S1: Sensitivity analysis using narrower histological eligibility criteria; Table S2: Exploratory thresholds selected by the Youden index; Table S3: Distribution of recorded histological descriptions; Table S4: Characteristics according to data availability. The supplementary tables are provided in the accompanying supplementary file.
