# Supplementary material for: Evaluation of Regional Body Circumferences and Serum Albumin for Identifying Nutritional Risk in Gastric Adenocarcinoma
Source: Medicina (Kaunas). 2026 Oct 19;62(10):2023. doi: 10.3390/medicina62102023 (PMC13707264; doi:10.3390/medicina62102023)
Supplement: Supplementary file 1 [file medicina-62-02023-s001.zip › medicina-4608948-supplementary.pdf]

*Supplementary Materials*

# Evaluation of Regional Body Circumferences and Serum Albumin for Identifying Nutritional Risk in Gastric Adenocarcinoma

**Oğuzhan Kesen, Neşe Alyıldız, Esra Aydın, Sıtki Utku Akay, and Ali Çelik**

**Table** S1.Sensitivity analysis using narrower histological eligibility criteria

**(A) AUC estimates in the common sample**

| Measure | AUC (95% CI) |
|---|---|
| Arm circumference | 0.860(0.765-0.956) |
| Abdominal circumference | 0.774(0.658-0.890) |
| Thigh circumference | 0.813(0.690-0.937) |
| BMI | 0.864(0.779-0.950) |
| Albumin | 0.882(0.810-0.953) |

**(B) Mutually adjusted associations in the arm plus albumin model**

| Term | OR (95% CI) | P |
|---|---|---|
| Arm, per 1-cm decrease | 1.56(1.15-2.12) | 0.004 |
| Albumin, per 0.1-g/dL decrease | 1.35(1.10-1.65) | 0.004 |

**(C) Model performance**

| Metric | Arm only Apparent | Arm only Corrected | Arm plus albumin Apparent | Arm plus albumin Corrected |
|---|---|---|---|---|
| AUC | 0.860 | 0.859 | 0.936 | 0.929 |
| Brier score | 0.103 | 0.109 | 0.084 | 0.094 |
| Calibration slope | 1.000 | 0.991 | 1.000 | 0.923 |
| Calibration intercept | 0.000 | 0.020 | 0.000 | -0.010 |

This analysis is restricted to patients with an explicit adenocarcinoma diagnosis: 92 patients overall, 90 with NRS-2002 data, and 18 with NRS-2002 ⩾3 (20.0%; 95% CI, 12.3–29.8%). All panels use the same 86 complete cases, including 17 with NRS-2002 ⩾3.

Paired AUC differences: arm minus BMI, −0.004 (95% CI, −0.075 to 0.066); arm minus albumin, −0.021 (−0.139 to 0.096). Both Holmadjusted p = 1.000. The likelihood-ratio test for adding albumin gave $\chi^2 = 12.13$, df = 1, p <0.001.

AUC CIs use DeLong; OR CIs use Wald. Panel B p values are unadjusted. AUC, area under the receiver operating characteristic curve; CI, confidence interval; OR, odds ratio. BMI, body mass index; NRS-2002, Nutritional Risk Screening 2002. The sensitivity cohort is a subset of the main cohort and does not provide external validation.

**Table S2.** Exploratory thresholds selected by the Youden index

**(A) Main cohort**

| Measure | Threshold | n | Sensitivity,% | Specificity,% | PPV,% | NPV,% |
|---|---|---|---|---|---|---|
| Arm circumference, cm | <22.5 | 100 | 55.0 | 96.3 | 78.6 | 89.5 |
| Abdominal circumference, cm | <81.5 | 100 | 65.0 | 80.0 | 44.8 | 90.1 |
| Thigh circumference, cm | <37.5 | 100 | 60.0 | 90.0 | 60.0 | 90.0 |
| BMI, kg/m2 | <22.34 | 100 | 90.0 | 71.3 | 43.9 | 96.6 |
| Albumin, g/dL | <3.615 | 95 | 94.7 | 69.7 | 43.9 | 98.1 |

**(B) Histology sensitivity analysis**

| Measure | Threshold | n | Sensitivity,% | Specificity,% | PPV,% | NPV, % |
|---|---|---|---|---|---|---|
| Arm circumference, cm | <22.5 | 90 | 55.6 | 95.8 | 76.9 | 89.6 |
| Abdominal circumference, cm | <81.5 | 90 | 61.1 | 81.9 | 45.8 | 89.4 |
| Thigh circumference, cm | <37.5 | 90 | 61.1 | 91.7 | 64.7 | 90.4 |
| BMI, kg/m2 | <22.34 | 90 | 88.9 | 72.2 | 44.4 | 96.3 |
| Albumin, g/dL | <3.615 | 86 | 100.0 | 69.6 | 44.7 | 100.0 |

Values below each threshold were classified as NRS-2002 ≥3. Thresholds maximize the Youden index. Sensitivity, specificity, PPV, and NPV are apparent estimates from the same samples used for threshold selection.

Main cohort: anthropometric measures,n = 100 with 20 NRS-2002 ≥3; albumin, n=95 with 19 NRS-2002 ≥3. Histology sensitivity analysis: anthropometric measures, n = 90with 18 NRS-2002 ≥3; albumin, n=86with 17 NRS-2002 ≥3.

Thresholds are rounded for presentation; performance estimates use unrounded thresholds. BMI, body mass index; PPV, positive 24predictive value; NPV, negative predictive value.

Table S3. Distribution of recorded histological descriptions

| Recorded description | n | % | Explicit adenocarcinoma | Compatible description |
|---|---|---|---|---|
| Adenocarcinoma without a specified subtype | 67 | 65.7 | 67 | 0 |
| Carcinoma with signet-ring cell features | 16 | 15.7 | 10 | 6 |
| Intestinal type | 6 | 5.9 | 4 | 2 |
| Diffuse type without an explicit signet-ring description | 3 | 2.9 | 2 | 1 |
| Mixed or tubular with poorly cohesive features | 2 | 2.0 | 2 | 0 |
| Mucinous adenocarcinoma | 2 | 2.0 | 2 | 0 |
| Poorly cohesive carcinoma | 2 | 2.0 | 1 | 1 |
| Poorly differentiated adenocarcinoma | 2 | 2.0 | 2 | 0 |
| Tubular or tubular-solid adenocarcinoma | 2 | 2.0 | 2 | 0 |
| Total | 102 | 100.0 | 92 | 10 |

Categories summarize the terminology recorded for the 102 patients and are mutually exclusive for reporting; they do not represent a centrally reassessed WHO or Lauren classification. Signet-ring descriptions take precedence over other descriptors. Tubular carcinoma with poorly cohesive features is included in the mixed group. The unspecified category includes records describing adenocarcinoma without a subtype, including adenocarcinoma in a metastatic sample; the wording alone was not used to infer disease stage.

The explicit adenocarcinoma column identifies the 92 patients retained in the narrower sensitivity cohort. The 10 compatible descriptions were six signet-ring, two intestinal, one diffuse, and one poorly cohesive description without an explicit adenocarcinoma term.

Table S4. Characteristics according to data availability

(A) Common complete-case analysis

| Characteristic | Included($n = 95$) | Not included($n = 7$) |
|---|---|---|
| Age, years | 58.0(49.5 − 66.0); n = 95 | 65.0(59.0 − 66.0); n = 7 |
| Male sex | 70/95(73.7%) | 5/7(71.4%) |
| BMI, kg/m2 | 22.9(20.1 − 26.9); n = 95 | 23.0(20.8 − 24.7); n = 7 |
| Arm circumference, cm | 26.0(24.0 − 28.0); n = 95 | 27.0(26.0 − 28.5); n = 7 |
| Abdominal circumference, cm | 87.0(81.0 − 97.5); n = 95 | 88.0(81.5 − 94.5); n = 7 |
| Thigh circumference, cm | 42.0(38.0 − 45.0); n = 95 | 42.0(40.0 − 44.0); n = 7 |
| Albumin, g/dL | 3.73(3.34 − 4.04); n = 95 | 4.12(4.04 − 4.21); n = 2 |
| NRS-2002 total score | 2.0(1.0 − 2.0); n = 95 | 2.0(1.0 − 2.0); n = 5 |
| NRS-2002 ≥3 | 19/95(20.0%) | 1/5(20.0%) |
| Three-month weight loss, kg | 6.0(5.0 − 10.0); n = 61 | 10.0 (10.0-13.8);n = 4 |

Continuous values are medians (interquartile ranges); n gives the available number for each variable. Categorical values are numerator/available denominator (%). The common sample required NRS-2002 and all five measures. Two patients lacked NRS-2002and five additional patients lacked albumin. No patient lacked the other main analysis measurements. Weight loss was not required for the main model. BMI, body mass index; NRS-2002, Nutritional Risk Screening 2002.

Table S4. Characteristics according to data availability continued

(B) Three-month weight-loss information

| Characteristic | Available($n$ = 65) | Missing($n$ = 37) |
|---|---|---|
| Age, years | 59.0(49.0 − 67.0); n = 65 | 58.0(52.0 − 64.0); n = 37 |
| Male sex | 45/65(69.2%) | 30/37 (81.1%) |
| BMI, kg/m2 | 22.5(19.0 − 25.3); n = 65 | 25.6(22.0 − 27.4); n = 37 |
| Arm circumference, cm | 25.0(23.0 − 28.0); n = 65 | 27.0(24.0 − 30.0); n = 37 |
| Abdominal circumference, cm | 87.0(80.0 − 95.0); n = 65 | 95.0(83.0 − 102.0); n = 37 |
| Thigh circumference, cm | 42.0(38.0 − 45.0); n = 65 | 43.0(40.0 − 46.0); n = 37 |
| Albumin, g/dL | 3.60(3.21 − 3.98); n = 63 | 3.89(3.69 − 4.30); n = 34 |
| NRS-2002 total score | 2.0(1.0 − 3.0); n = 63 | 1.0(1.0 − 1.0); n = 37 |
| NRS-2002 23 | 19/63(30.2%) | 1/37(2.7%) |
| Three-month weight loss, kg | 7.0(5.0 − 10.0); n = 65 | Not available |

Continuous values are medians (interquartile ranges); n gives the available number for each variable. Categorical values are 43numerator/available denominator (%). Two patients with recorded weight loss lacked NRS-2002; consequently, the nutritional-risk denominator is 63 in that group. A missing separate weight-loss field does not establish that weight loss was unavailable at the original clinical screening. The recorded NRS-2002 total wvas used as documented. BMI, body mass index; NRS-2002, Nutritional Risk Screening 2002.

Table S4. Characteristics according to data availability continued

(C) Serum albumin

| Characteristic | Available($n$ = 97) | Missing($n$ = 5) |
|---|---|---|
| Age, years | 58.0(50.0 − 66.0); n = 97 | 66.0(65.0 − 66.0); n = 5 |
| Male sex | 72/97(74.2%) | 3/5(60.0%) |
| BMI, kg/m2 | 22.9(20.1 − 26.8); n = 97 | 23.0(20.1 − 24.1); n = 5 |
| Arm circumference, cm | 26.0(24.0 − 28.0); n = 97 | 28.0(25.0 − 29.0); n = 5 |
| Abdominal circumference, cm | 88.0(81.0 − 97.0); n = 97 | 83.0(80.0 − 95.0); n = 5 |
| Thigh circumference, cm | 42.0(38.0 − 45.0); n = 97 | 42.0(38.0 − 42.0); n = 5 |
| Albumin, g/dL | 3.74(3.34 − 4.05); n = 97 | Not available |
| NRS-2002 total score | 2.0(1.0 − 2.0); n = 95 | 2.0(1.0 − 2.0); n = 5 |
| NRS-2002 23 | 19/95(20.0%) | 1/5(20.0%) |
| Three-month weight loss, kg | 7.0(5.0 − 10.0); n = 63 | 10.0(10.0 − 10.0); n = 2 |

Continuous values are medians (interquartile ranges); n gives the available number for each variable. Categorical values are 50numerator/available denominator (%). BMI, body mass index; NRS-2002, Nutritional Risk Screening 2002.

Table S4. Characteristics according to data availability continued

(D) NRS-2002total score

| Characteristic | Available($n$ = 100) | Missing($n$ = 2) |
|---|---|---|
| Age, years | 58.5(50.0 − 66.0); n = 100 | 59.0(58.0 − 60.0); n = 2 |
| Male sex | 73/100(73.0%) | 2/2(100.0%) |
| BMI, kg/m2 | 23.0(20.1 − 26.8); n = 100 | 23.4(22.4 − 24.4); n = 2 |
| Arm circumference, cm | 26.0(24.0 − 28.0); n = 100 | 27.0(27.0 − 27.0); n = 2 |
| Abdominal circumference, cm | 87.0(80.8 − 97.2); n = 100 | 91.0(89.5 − 92.5); n = 2 |
| Thigh circumference, cm | 42.0(38.0 − 45.0); n = 100 | 44.0(43.0 − 45.0); n = 2 |
| Albumin, g/dL | 3.73(3.34 − 4.04); n = 95 | 4.12(4.04 − 4.21); n = 2 |
| NRS-2002 total score | 2.0(1.0 − 2.0); n = 100 | Not available |
| NRS-2002 ≥3 | 20/100 (20.0%) | Not available |
| Three-month weight loss, kg | 7.0(5.0 − 10.0); n = 63 | 17.5(13.8 − 21.2); n = 2 |

Continuous values are medians (interquartile ranges); n gives the available number for each variable. Categorical values are 54numerator/available denominator (%). BMI, body mass index; NRS-2002, Nutritional Risk Screening 2002.
